# The FIGS (Focused Identification of Germplasm Strategy) Approach Identifies Traits Related to Drought Adaptation in *Vicia faba* Genetic Resources

**DOI:** 10.1371/journal.pone.0063107

**Published:** 2013-05-08

**Authors:** Hamid Khazaei, Kenneth Street, Abdallah Bari, Michael Mackay, Frederick L. Stoddard

**Affiliations:** 1 Department of Agricultural Sciences, University of Helsinki, Helsinki, Finland; 2 International Centre for Agricultural Research in the Dry Areas, Aleppo, Syria; 3 Queensland Alliance for Agricultural and Food Innovation, The University of Queensland, St Lucia, Queensland, Australia; CIRAD, France

## Abstract

Efficient methods to explore plant agro-biodiversity for climate change adaptive traits are urgently required. The focused identification of germplasm strategy (FIGS) is one such approach. FIGS works on the premise that germplasm is likely to reflect the selection pressures of the environment in which it developed. Environmental parameters describing plant germplasm collection sites are used as selection criteria to improve the probability of uncovering useful variation. This study was designed to test the effectiveness of FIGS to search a large faba bean (*Vicia faba* L.) collection for traits related to drought adaptation. Two sets of faba bean accessions were created, one from moisture-limited environments, and the other from wetter sites. The two sets were grown under well watered conditions and leaf morpho-physiological traits related to plant water use were measured. Machine-learning algorithms split the accessions into two groups based on the evaluation data and the groups created by this process were compared to the original climate-based FIGS sets. The sets defined by trait data were in almost perfect agreement to the FIGS sets, demonstrating that ecotypic differentiation driven by moisture availability has occurred within the faba bean genepool. Leaflet and canopy temperature as well as relative water content contributed more than other traits to the discrimination between sets, indicating that their utility as drought-tolerance selection criteria for faba bean germplasm. This study supports the assertion that FIGS could be an effective tool to enhance the discovery of new genes for abiotic stress adaptation.

## Introduction

Drought coupled with heat stress, expected to increase in frequency and intensity is likely to expand due to climate change [1], [2]. Faba bean (*Vicia faba* L.) is an important source of protein, often referred to as poor man’s meat, in those dry areas of developing countries most likely to be impacted by climate change [3], [4]. This has significant food security implications because faba bean is relatively sensitive to terminal moisture stress when compared to other temperate-season grain legumes [5]–[7] so drought is a major constraint to its production and yield stability. Therefore it is imperative that natural variation for traits related to drought adaptation be identified from the faba bean genepool and introduced into improved cultivars. Economic analysis of cultivar development showed that the identification of a desirable trait is of equal importance to the process of transferring it into improved backgrounds because it reduces the time taken to develop improved cultivars [8].

Genetic resource collections conserved in genebanks are the most obvious place to look for useful traits, but given the size of these collections, searching for specific and often rare traits has been likened to searching for a needle in a haystack. Further, evaluating large collections for some parameters can be extremely costly. For example, the International Center for Agricultural Research in the Dry Areas (ICARDA) houses a globally important collection of over 9500 faba bean accessions. It would be beyond the resources of most research programs to evaluate this entire collection for variation in leaf morpho-physiological traits related to plant moisture stress. What is needed therefore is a means of wisely selecting an economically feasible set size that has a better probability of capturing useful variation than if material was selected at random or through the use of other techniques that do not focus on the sought-after trait.

The core collection was proposed as a way to work with fewer accessions that would represent, “with a minimum of repetitiveness, the genetic diversity of a crop species and its relatives” [9]. There are numerous examples of methodologies to develop core collections (*see* Hodgkin et al. [10] for examples), which in practice tend towards limiting the size of the sub-set to around 10% [11], [12] of the original collection size. Although one of the stated purposes of core collections is to improve utilization, the vast majority of reported research seems to focus more on methods (or sampling strategies) to establish core collections [13]–[16] and the analysis of the diversity held within core collections [17]–[20]. A number of references suggest alternative types of collections, or sets of collections, to enhance the efficiency of capturing diversity or addressing utilization, including specialized core collections [21], mini core sets [22], nested core collections [23] and composite collections [24]. Despite this diversity of core collection methodology, there seems to be a lack of literature that demonstrates that core collections have had a significant impact on the utilization of genetic resources. Rare and adaptive alleles, most of which are thought to be functional, may even be missed from a core collections [21], [25]–[29].

The Focused Identification of Germplasm Strategy (FIGS) was designed to improve the efficiency with which specific adaptive traits are identified from genetic resource collections. FIGS is based on the premise that adaptive traits displayed by an accession will reflect the selection pressures of the environment from which it was originally sampled [30]–[33]. The FIGS approach uses both trait and environmental (climate) data to develop *a priori* information or specialized knowledge as per Gollin et al. [8] based on a quantification of the trait-environment relationship [32], [34], [35]. This *a priori* information is then used to define a set of accessions with a high probability of containing the desired traits.

Many adaptive traits can be linked to agro-climatic parameters. For example, using monthly values for a range of climatic variables, FIGS detected sources of resistance in wheat (*Triticum* ssp.) for biotic stresses such as powdery mildew (*Blumeria graminis* (DC) Speer f.sp. *tritici*) [36], [37], Sunn pest (*Eurygaster intergriceps* Put.) [38], Russian wheat aphid (*Diuraphis noxia* Kurdj.) [39] and stem rust (*Puccinia graminis* Pers.) [34], [35] as well as net blotch (*Pyrenophora teres* Drechs.) in barley (*Hordeum vulgare* L.) [34]. Further, Endresen et al. [40] demonstrated how eco-geographic data from the collection sites of 14 Nordic barley landraces (*Hordeum vulgare* L.) was successfully correlated to morphological traits using multilinear data modelling techniques and conclude that the FIGS approach can be used efficiently as a targeted germplasm selection method.

However, so far studies are few on the effectiveness of FIGS to detect traits that impart tolerance to abiotic stresses such as moisture availability, and there are certainly none for faba bean.

The aim of this study was to compare the leaf morpho-physiology and phenology of two sets of faba bean accessions originating from environments with contrasting seasonal moisture availabilities. The underlying hypothesis is that ecotypic (climatic) differentiation occurred so traits associated with plant moisture regulation and lifecycle will differ between the two sets. From this, we would further expect that set membership based on collection site environmental descriptors would be the same when the accessions are classified using trait measurements.

## Materials and Methods

### Construction of FIGS Sets

**Figure 1:**
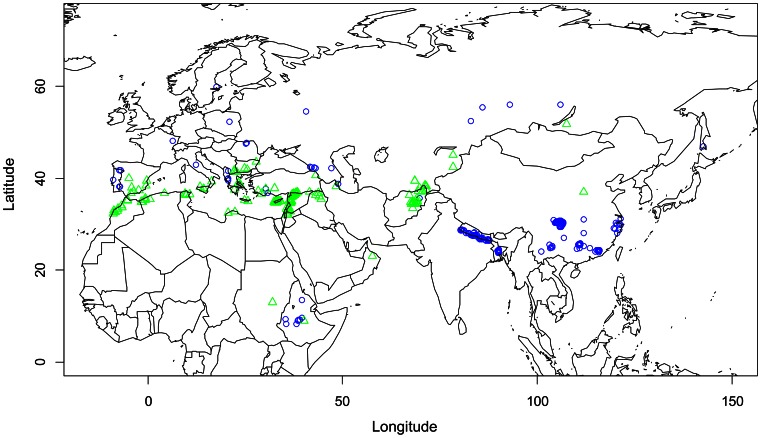
Geographical distribution of the two sets (wet set, blue circle and dry set, green triangle).

Two sets containing landrace accessions of faba bean were selected from the collection conserved by ICARDA that contains 9545 entries, representing 21% of the worldwide germplasm collection [41]. One set was chosen to maximize the probability of having drought-related adaptive traits, the “dry set”, and the other was constructed as a control from accessions originating from environments with higher moisture profiles - the “wet set”. The origin of the selected accessions is presented in Figure 1, and the ICARDA accession numbers are given in Table S1.

The dry set (201) was constructed as follows. Accessions from collection sites where the annual rainfall was below 300 mm/year or greater than 550 mm/year were not considered. Of the remaining accessions, one accession per collection site was chosen at random. A hierarchical cluster analysis was performed using the following collection site agro-climatic parameters: *precyr, ariyr*, *tminyr, tmaxyr, bio4*, *bio15, bio16, and bio19* extracted from ICARDA and Worldclim-databases and Hijmans et al. [42] (Table 1). The climate variables were chosen to combine temperature and precipitation factors that would influence the length of growing season and seasonal moisture availability. The between-groups linkage option was set as the clustering algorithm, using squared euclidian distances as the distance measure. The procedure created 20 clusters. Accessions contained in 6 clusters were dropped because the average aridity index for the cluster was above 0.6 or below 0.1 (indicating irrigated sites). For each of the remaining clusters the accessions were sorted according to the *bio15* climate variable (a measure of the variation in seasonal moisture availability) for their respective collection sites. Any accession with a score of 50 or lower was discarded. The remaining accessions within each cluster were ranked based on collection site long-term yearly precipitation. A set of 201 accessions was chosen by selecting the lowest ranked accession in each cluster and repeating the process until the set size was achieved.

**Table 1:** The climatic variables used in the selection of FIGS sets.

Code	Description
*precyr*	Long term yearly precipitation
*ariyr*	Long term yearly aridity index
*tminyr*	Long term yearly minimum temperature
*tmaxyr*	Long term yearly maximum temperature
*Bio4*	Temperature seasonality (standard deviation × 100)
*Bio15*	Precipitation seasonality (coefficient of variation)
*Bio16*	Precipitation of wettest quarter
*Bio19*	Precipitation of coldest quarter

Source: ICARDA GIS (Geographic information system) unit and world global climate data (http://www.worldclim.org/bioclim).

The wet set was chosen from sites that receive over 800 mm/year of rainfall (long-term average). One accession per site was chosen at random. The remaining accessions were sorted according to collection site yearly average aridity index and 201 accessions were chosen from sites with the highest aridity indices.

### Growth Conditions

Accessions were planted in a randomized complete block design (RCBD) with 4 replicates in a climate-controlled greenhouse of the University of Helsinki, Finland during 2010–2011, giving a total of 1608 pots. Before sowing, seeds were inoculated with *Rhizobium leguminosarum* biovar. *viciae* (faba bean strain, Elomestari Oy, Tornio, Finland). Three seeds were sown per 2 L plastic pot, which held a mixture of sand and peat (White 420 W, Kekkilä Oy, Vantaa, Finland) (3∶1 *v/v*). After 10 days, the seedlings were thinned to one per pot. Soil moisture levels were maintained at field capacity with an automatic irrigation system to ensure that each plant received the same amount of water during the experiment. At three and five weeks after sowing, 70 ml of fertilizer solution (equivalent to 20 kg of P and 24 kg of K per hectare) was added to each pot. The photoperiod was adjusted to 14 h light and 10 h dark, and the temperature was set to 21°C day/15°C night ±2°C. Photosynthetic photon flux density (PPFD) was about 300 µmol m^–2^ s^–1^ at the canopy level. The relative humidity was maintained at 60±5%.

#### Pest control

Thrips were controlled biologically using *Amblyseius cucumeris,* especially at seedling and flowering stages.

### Morphological Measurements

#### Stomatal density and morphology

Stomatal density (SD), length (SL) and width (SW) were measured on the middle part of the abaxial surface of the youngest, fully expanded leaflet of 8-week-old plants using the impression method [43]. The number of stomata was counted from ten different microscopic fields of view at 250× magnification. To estimate SD, the number of stomata per field of view was converted to the number of stomata per mm^2^ of leaf using a standard scale. SL and SW were measured on ten stomata from the impressions using a scaled 500× eyepiece of microscope and converted to µm. Stomatal area (SA) was calculated as SA = SL × SW. Stomatal area per unit area of leaflet (SAAL) was calculated as the product of SA and SD.

#### Leaflet area

Leaflet area was measured using a LI-6200 leaf area meter (LI-COR, Inc., Lincoln, NE, USA). Means of four leaflets per plant were used for analysis.

#### Fertile tillers

The number of fertile tillers was counted at 16 weeks after sowing.

#### Seed size

Ten seeds from each accession were measured in order to classify them to the traditional seed size class, *minor*, *equina* and *major* according to seed length and mass [44], [45].

### Physiological Measurements

#### Gas exchange traits

Gas exchange was measured on each plant at 6 weeks and 8 weeks after sowing, using a LI-6400 portable photosynthesis system (LI-COR, Inc.) equipped with a 2×3 cm leaf chamber with a LED light source (6400-02B, 90% red and 10% blue). Photosynthesis photon flux density (PPFD) was 1000 µmol m^−2^ s^−1^. A CO_2_-injecting cartridge was attached to the system to control reference CO_2_ concentration at 400 µmol mol^−1^, a value close to that during plant growth. The flow rate was 400 µmol s^−1^. All the gas exchange measurements were done between 9 and 11 am using the youngest, fully expanded leaflet which was also used for stomatal morphology and leaflet area measurements. Measurements were logged only when the stability criteria were met, according to the manufacturer’s instructions. For logistical reason, each replicate was measured on a separate day. The gas exchange measurements taken were: photosynthetic rate (A_net_), stomatal conductance (g_s_), transpiration rate (E), and intercellular CO_2_ (C_i_). Intrinsic water use efficiency (WUE) was calculated as gas exchange rate divided by stomatal conductance (A_net_/g_s_) [46].

#### Leaflet and canopy temperatures

Leaflet temperature was measured along with gas exchange on the LI-6400. Canopy temperature was measured using a FLUKE® 574 thermometer gun (FLUKE, Everett, WA, USA) from the fully expanded leaves used for the other measurements. Canopy temperate was measured at 6 weeks and 8 weeks after sowing. Air temperature was recorded at the time of measuring leaf temperature. Leaflet temperature is presented as: Leaflet temperature – air temperature and canopy temperature as: canopy temperature – air temperature.

#### Relative water content

Five leaflets were used for determining leaf relative water content (RWC%) according to the initial principles by Barrs and Weatherley [47]. First, fresh weight (FW) was determined. Turgid weight (TW) was measured after floating the sample on distilled water in Petri dishes in darkness at 4°C for 24 h. Dry weight (DW) was calculated by putting the samples for 48h in a 60°C oven. RWC (%) = (FW–DW)/(TW–DW) × 100.

### Phenological Measurement

The number of days to the onset of flowering was recorded.

### Statistical Analysis

The membership of the two contrasting FIGS sets was based on *a priori* information, namely the long-term climatic conditions of the sites from which the accessions were collected. The underlying assumption was that morpho-physiological traits related to moisture stress adaptation would differ between two sets of selected germplasm. Two methods were used to determine whether the two sets are different in terms of morpho-physiological phenotypic expression.

To determine if there were differences between the sets, they were subjected to a t-test, using means across replicates for each accession, with the R statistical package [48] after testing for normality.

Multivariate analysis was employed for deeper investigation because the relationships between the collection site agro-climatic conditions and trait expression are likely to be non-linear and multi-dimensional and thus not captured in a linear framework. When trait expression differs between the two sets, this should be reflected in how the classification algorithms discriminate between accessions. Thus, we would expect the algorithms to correctly assign accessions into the sets created on climatic descriptors. Three models (Table 2) were used to classify accessions, discriminate between sets and to highlight those traits that contributed most to the discrimination. The algorithms used a learning-based approach, in which they were “trained” on a set of accessions whose set membership (wet or dry) was made “known” to the algorithm. The trained algorithm was then used to classify the accessions whose set membership was “unknown” to the algorithm into two sets (wet or dry). This is an iterative process where the model that is finally chosen by the algorithm is based on the “best” values for accuracy parameters that measure the model’s ability to classify the unknown accessions into their respective climate-based sets. These learning-based techniques need fewer assumptions and thus are more suitable when highly complex non-linear relationships are expected among input variables. They were used to overcome the problem of restrictive parametric paradigms on one hand and the prerequisite distribution assumptions on the other [56], [57].

**Table 2:** Models used in the study to test the difference between the two sets and to select the best splitters.

Model	Tuning parameters	Library(R language)	References
Classification and Regression Training (CARET)*		caret	[49], [50]
Random Forests (RF)*	Number of trees (n.tree)Number of predictors chosen ateach node (mtry)	randomForest	51–53
Support Vector Machines(SVM)	gamma/sigma, cost (C)	svm (e1071)ksvm (kernalab)	[49], [54], [55]

* Variable importance is available for these models.

The parameters used to measure the accuracy of these models are the AUC and Kappa values. The AUC refers to the area under the curve (AUC) of the Receiver Operating Characteristics (ROC) [58], [59], which is a plot of true positive rate versus false positive [60]. An AUC value of 0.5 represents randomness and would indicate that the FIGS sets are no different from randomly chosen sets. An AUC value of 0.7 and above represents high model performance [59] indicating that the wet and dry sets are highly distinguishable and that the dry set is more prone to harbor traits that favor drought adaptation. Similar to the AUC, Kappa is a measure of agreement, where a value of 0.4 and above is an indication of good agreement between the model’s prediction and the trait measurements [61].

The datasets were presented to the algorithms as follows: the mean value for each variable was calculated over the replicates for each accession. This accession level data was combined (wet+dry sets) and standardized so that the dataset mean was zero with standard deviation of 1. The algorithms split the combined data into 2 datasets containing 2/3 and 1/3 of the accessions on a random basis. The larger dataset was used to “train” the models and quantify the association between the membership (wet/dry) and the drought-related attributes. The association was then used in turn (in reverse) to classify the “unknown” accessions of the smaller dataset. This process was performed 10 times and the results were averaged.

#### Selection of important parameters

Some of the parameters used to differentiate between two sets are expected to have more influence on the classification defined by the algorithms (Table 2). The importance of each variable was calculated based on the Gini, or impurity index, where a split node that has a mixture of both tolerance and susceptible membership (wet and dry set) is less pure.

## Results

Eleven of the 16 parameters measured differed between the sets. The members of the dry set had 21% fewer fertile tillers, flowered 2.4 days earlier, had longer stomata (4%), greater stomatal area (4%), more stomatal area per unit of leaflet (3%), 48% more leaflet area, 5% higher transpiration rate, 5% higher RWC, and cooler leaves than the wet set. The transpiration rate was 9% higher in the wet set while leaflet and canopy temperatures were lower in the dry set (Table 3). Furthermore, three quarters of the material from the dry set were large-seeded (*major* type) compared to only 20% in the wet set, whose remaining seeds were distributed equally between the *minor* and *equina* classes (Figure S1). The two sets thus contained accessions that, on average, differed morphologically and physiologically.

**Table 3:** The mean (± standard deviation) of morphological, physiological and phenological measurements on sets of 201 wet adapted and 201 dry adapted faba bean accessions, along with the difference between the set means and the value of the t-test.

Parameters	means		*difference*	t _(df = 400)_
	Wet	Dry		
***Morphology***				
Stomatal density *(No. mm* ^−*2*^ *)*	**49.6**±12.4	**48.3**±6.1	–1.3	1.29^ns^
Stomatal length *(µm)*	**53.2**±3.9	**55.4**±2.6	+2.2	6.63^***^
Stomatal width *(µm)*	**30.4**±1.61	**30.3**±0.92	–0.1	0.20^ns^
Stomatal area *(µm^2^)*	**1622**±184	**1685**±119	+63	4.07^***^
Stomatal area per unit area of leaflet × 10^−3^ *(µm^2^.mm* ^−*2*^ *)*	**77.9**±12.4	**80.6**±6.8	+2.7	2.67^**^
Leaflet area *(cm^2^)*	**11.4**±5.9	**16.9**±5.2	+5.5	9.86^***^
Number of fertile tillers	**2.85**±1.52	**2.25**±1.1	–0.60	4.54^***^
***Physiology***				
Photosynthetic rate *(µmol m* ^−*2*^ * s* ^−*1*^ *)*	**7.8**±2.0	**8.2**±1.1	+0.4	2.25^*^
Intercellular CO_2_ *(ppm)*	**322.4**±22.3	**325.2**±10.5	+2.8	1.64^ns^
Stomatal conductance *(mol m* ^−*2*^ * s* ^−*1*^ *)*	**0.329**±0.119	**0.320**±0.072	–0.009	0.92^ns^
Water use efficiency *(µmol mol* ^−*1*^ *)*	**25.6**±7.8	**26.4**±4.2	+0.8	1.24^ns^
Transpiration rate *(mmol m* ^−*2*^ * s* ^−*1*^ *)*	**3.26**±0.9	**3.42**±0.5	+0.16	2.15^*^
Leaflet temperature *(°C)*	**–0.17**±0.31	**–0.72**±0.19	–0.55	34.98^***^
Canopy temperature *(°C)*	**–0.80**±1.21	**–1.59**±0.48	–0.79	13.77^***^
Relative water content *(%)*	**82.3**±3.9	**86.3**±2.4	+4.0	12.59^***^
***Phenology***				
Days to flowering	**50.7**±13.1	**48.3**±7.4	–2.4	2.24^*^

df: degrees of freedom; ns: non significant; *, *P*<0.05; **, *P*<0.01; ***, *P*<0.001.

This assertion is supported by all 3 models used to classify the accessions based on the trait data; the accessions were placed into sets that agreed with the original climate-based classifications. The Kappa scores were all close to one, which demonstrates a high degree of accuracy given that an acceptable score is above 0.4. Likewise the AUC values were well in excess of the acceptable value of 0.7. Thus the models classified the accessions into their climate-based sets with accuracies approaching 100% (Table 4).

**Table 4:** Model accuracy values for learning-based techniques used on test data (1/3) of faba bean over 10 runs of the algorithms.

Model		AUC	omission rate	sensitivity	specificity	correct classificationrate	Kappa
caret-rpart	**Mean**	**0.97**	**0.03**	**0.97**	**0.97**	**0.97**	**0.93**
	Lower	0.96	0.01	0.95	0.95	0.96	0.92
	Upper	0.98	0.05	0.99	0.98	0.97	0.95
RF	**Mean**	**0.99**	**0.01**	**0.99**	**1.00**	**0.99**	**0.99**
	Lower	0.99	0.00	0.98	1.00	0.99	0.98
	Upper	1.00	0.02	1.00	1.00	1.00	1.00
SVM	**Mean**	**0.99**	**0.00**	**1.00**	**0.98**	**0.99**	**0.98**
	Lower	0.99	0.00	1.00	0.97	0.99	0.97
	Upper	1.00	0.00	1.00	0.99	1.00	0.99

AUC: Area under the ROC curve.

RF: Random Forest.

caret-rpart: Classification and Regression Training.

SVM: Support vector machine.

Correct classification rate: the overall classification of both wet and dry accessions to their respective membership group. It is the total of both correctly classified accessions as either wet or dry divided by the total of all the accessions (402).

Omission rates: the opposite of correctly classified accessions with drought-related traits in this case, which is the number of accessions lacking the traits yet they have been classified (incorrectly) as having the.

**Figure 2:**
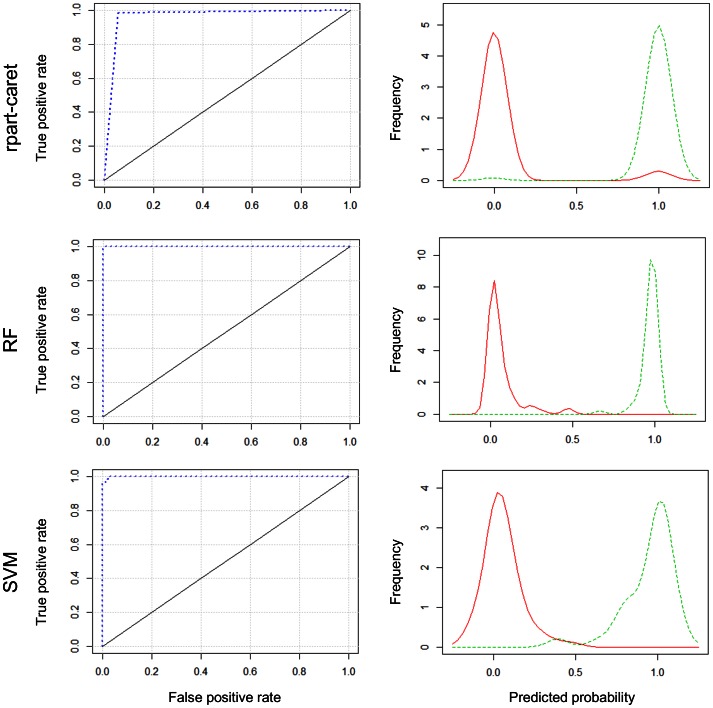
ROC plots (left) and density plots class prediction (right) for dry and wet sets using the three models; The class predictions fall out of range (0, 1) as a result of linearity/interpolation in some of the models.

The accuracy of the models is also illustrated by the ROC plots (Figure 2), where displacement above the diagonal indicates non-random assignment of accessions to the correct subset. In the rpat-caret plot, there is some overlap between the sets, but both RF and SVM show mutual exclusivity of the two sets. The prediction density plots to the right of the ROC plots demonstrate that the wet and dry sets include accessions which, in a multivariate sense, are different and that the basis for the difference will be related to the selection criteria, in this case the seasonal moisture availability at collection sites.

### Variable Importance

Of the 16 variables, leaflet temperature depression was the most informative, followed by canopy temperature, RWC, leaflet area and stomatal length (Table 5). The relative importance of the other variables differed between the three assessment methods, with transpiration rate being the third most important in RF mean decrease accuracy and fourth in RF mean decrease Gini, for example.

**Table 5:** Potential climate predictors based on caret R and RF packages.

Rank	drought related parameter	model			
		rpart-caret	RF		
			mean decrease accuracy		mean decrease Gini
**1**	**Leaflet temperature**	**34.91**	**0.26**	**55.41**	
**2**	**Canopy temperature**	**13.68**	**0.10**	**31.64**	
**3**	**Relative water content**	**12.46**	**0.02**	**9.64**	
4	Leaflet area	9.95	0.01	6.39	
5	Stomatal length	6.70	0.01	2.30	
6	Fertile tillers	4.72	0.00	0.47	
7	stomatal area	4.13	0.00	1.01	
8	Transpiration rate	3.61	0.03	6.94	
9	Stomatal area per unit area of leaflet	2.75	0.01	3.52	
10	Photosynthetic rate	2.34	0.01	4.25	
11	Days to flowering	2.21	0.00	1.95	
12	Intercellular CO_2_	1.64	0.00	0.93	
13	Stomatal density	1.26	0.01	2.42	
14	Water use efficiency	1.21	0.01	1.89	
15	Stomatal conductance	0.86	0.01	1.83	
16	Stomatal width	0.14	0.00	0.82	

## Discussion

While other studies have shown that the FIGS approach was effective when employed in the search for resistance to pests and diseases (*e.g.* [34], [35], [37], [38]), this study demonstrates its effectiveness as a method to search for adaptive traits associated with abiotic constraints. The set selection process, based on indicators of moisture availability, yielded sets whose morpho-physiology and phenology were significantly different.

This result is not all that surprising, since it has been comprehensively shown that the environment strongly influences gene flow, natural selection and thus spatial/geographic differentiation [62]–[64]. Numerous studies have documented eco-geographic variation for drought-related traits linked to environmental parameters such as phenology and carbon isotope discrimination in *Triticum turgidum* spp. *dicoccoides* (Körn.) Thell [65], as well as leaf area, electrolyte linkage and RWC in *Arabidopsis thaliana* [66]. In this context, FIGS represents a logical extension of N. I. Vavilov’s work that by the 1920s had developed and illustrated the concept of centres of diversity that established the association between diversity and eco-geographic distribution [67].

Despite the above, using an eco-geographic approach to select germplasm for utilization has not been industry standard in genetic resource conservation circles. Rather, there has been a focus on the core collection concept (*e.g.* [21]). In fact, the Food and Agriculture Organization of the United Nations (FAO), in its global strategy for plant genetic resources (PGR) conservation, called for and financially supported the development of core collections as a standard and recommended practice.

However, the authors of this paper have determined that a large percentage of germplasm requests from the ICARDA genetic resources database are for specific adaptive traits. Thus it is argued that, in contrast to core collections, FIGS represents a dynamic, direct and practical approach that focuses on specific adaptive traits rather than on generalized measures of diversity, and as such could be of considerable value to the genebank user community if deployed on a regular basis. It is further suggested that as the plant breeding community prepare to tackle climate change, the efficient utilization of genetic resource collections will become increasingly important [68]. In this context, it is argued that the FIGS approach can reduce the cost and effectiveness of evaluation by reducing the number of accessions screened while providing a higher probability of identifying sought-after traits.

While this study supports the assertion that FIGS is an effective way to search for adaptive traits, there is considerable room for improvement in the approach. Since FIGS is still in its infancy, it is acknowledged that the procedure used to select the sets in this study was more a common sense process rather one based on previous research. The rationale behind the selection of the dry set was to select material from environments that were most likely to impose relatively dry conditions during the growing season whilst not so dry that a crop would need irrigation. Faba bean is unlikely to be planted to rain fed conditions much below the 300 mm/year limit. Further the criteria on narrow range in rainfall and low aridity index were selected to favour environments where there is more likely to be higher seasonal variation for moisture availability, low rainfall tending to be coupled with high variability. The rationale here is that higher seasonal moisture variation is likely to push populations towards physiological adaptation to dry conditions rather than drought avoidance strategies (earliness, for example). The *bio15* parameter, a measure of seasonal variation in rainfall, was then used to select high variation environments. The *tminyr, tmaxyr, bio4*, *bio16,* and *bio19* parameters were included in the clustering procedure because they all represent factors that influence growth conditions and it was desirable to include a range of different low-moisture environments. The approach outlined above to select the dry set could have been done in different ways and further experimentation is needed to determine the optimal strategy.

Different approaches could also have been used to define the set of material originating in environments with higher seasonal moisture profiles. In this case it was considered desirable to include a wide range of environments provided they received over 800 mm of precipitation, which is considered to be favourable for faba bean cultivation.

Both sets were chosen by applying selection criteria to long-term average yearly data. However, these data do not necessarily reflect the conditions within the growing season. A more effective approach would be to use climatic data presented on the basis of growing season or different crop development phases rather than calendar year. To do this effectively there is a need for accurate continuous surface maps detailing the onset of the growing season for different crop species. Additionally, the machine-learning algorithms used in this study could be used to create the FIGS sets using climatic variables as the input data.

While this study demonstrates that there is a difference in leaf morphology and physiology associated with water use between the two sets, it was performed under well watered conditions and thus we cannot firmly conclude that the dry set is in fact more drought tolerant. Nevertheless, the existence of a difference indicates that eco-typic differentiation has occurred in faba bean accessions from dryer environments, so we can infer that differentiation is in some way associated with adaptation to dryer seasonal moisture profiles. Indeed, eco-geographic differentiation has been found for leaf morphology in other species. For example leaf area was found to be negatively correlated with altitude (and by inference the probability of chilling stress) for *Dodonaea viscosa* subsp. *angustissima* [69]. It would appear that the same holds true for faba bean, since leaf width in this study was linked to maximum temperature regionally (latitude gradient) and leaf area to minimum temperature locally (altitude gradient).

While leaf area and RWC were positively correlated in *Quercus acutissima* [70], as found in this study (*R^2^* = 0.29, *P*<0.001, n = 402), leaf area and size diminished with declining water availability, in contrast to this study. The present results may be seen as somewhat counter-intuitive if one expects reduced leaf areas to present less evaporative surface, thus favouring tighter control on water use, which is certainly the case in xerophytic perennials. However, large leaf areas cover the soil surface more effectively, minimizing unproductive evaporation. Furthermore, 75% of the dry set accessions belong to the *major* seed type of faba bean (Figure S1) and these larger seeds tend to produce bigger seedlings with larger leaflets and more extensive root systems, which bestow the adaptive advantage of rapidly exploiting available soil moisture earlier in the season. In *Panicum virgatum* L., for example, larger seeds were linked to higher seedling vigour and better root establishment in dry environments [71], while in oat (*Avena sativa* L.) larger seeds lead to better germination under osmotic stress [72], and in faba bean larger seeds were related to higher transpiration efficiency and lower transpiration rates [73]. Furthermore, in some legume species seed size was found to be an indicator of abiotic adaptation [74].

RWC has been recognized as a reliable indicator of plant water status, and thus has been widely used as a screening parameter for drought adaptation in crop plants [47], [75]. Nevertheless, screening large quantities of germplasm using RWC measurements is costly and time consuming. Since lower RWC in this study was associated with lower canopy temperatures (*R^2^* = 0.54, *P*<0.001, n = 402), it supports the assertion of Blum [76] that leaf temperature can be used as a rapid and economical phenotyping method to screen germplasm for drought adaptation. The slightly earlier flowering in the dry set is in line with expectations that earlier flowering is part of drought escape in faba bean as in many other species [6].

The current work involved the aerial part of the plant. Nevertheless, for drought adaptation, root morphology and function also play a significant role [75], [77]. For example, the roots of sorghum genotypes from dry African environments were found to be deeper and more highly branched than US-derived genotypes [78]. Variation for root traits linked to drought adaptation is of particular interest, especially if they can be linked to more easily evaluated above-ground marker. A logical extension of the work reported here would be to assess differences in root morphology between the two sets.

Many genetic diversity studies still use linear based approaches such as principal component analysis (PCA). The machine learning/recursive algorithms used here represent a novel approach deserving some comment. This study demonstrates that the RF and SMV approaches are suited to studies such as this, since they can detect patterns or relationships between a dependent variable (trait data) and a set of independent variables (climate data) in large datasets [79]. They can also identify parameters that have the greatest impact on the discrimination. Used in this context, the algorithms can point to which trait or combination of traits confers the adaptation.

Further, the use of recursive partitioning is gaining momentum in areas where the data are too highly dimensional for standard regression methods such as PCA in which the decomposition of variables into reduced components leads to the loss of their individual effects, thus rendering the important variable unidentifiable in the interpretation [80]. In the present algorithms, the variables that have a strong relationship to the trait would be those that split the accessions correctly [81]. At the split, the variable that produces less entropy measured using either information theory (Shannon index) or Gini index (known as impurity measure) is ranked first. A reduction in the impurity is a prerequisite for the variable ranking/importance which can be best visualised in the graphs generated by these algorithms [82].

A further advantage of the algorithms used here is that the input data does not have to be normally distributed or conform to other assumptions related to linear models and thus do not require the tedious and time consuming pre-analysis required for linear models to ensure that the assumptions are not violated.

### Conclusions

The methods used were effective at creating sets that were different in terms of leaf morphology, physiology and phenology. This demonstrates that eco-geographic differentiation in faba beans has occurred and is related, in part, to moisture availability. Thus the underlying premise upon which FIGS is based was supported, indicating that it can be an effective tool to enhance the discovery and deployment of new genes, although the FIGS process can be improved to select for drought-adapted genetic resources. Further, the use of machine-learning algorithms was demonstrated here as an effective tool to investigate datasets that are complex and highly dimensional, so it is suggested that they are particularly suited to eco-geographic diversity studies. The results also indicate that leaf and canopy temperature could be an economical way to screen for potentially drought-adapted material as has been suggested by other authors.

## Supporting Information

Figure S1 **Distribution of seed size classes (** ***minor*** **,** ***equina*** **and** ***major*** **) among wet and dry set germplasm.**
(TIF)

Table S1 **List of ICARDA accession number (wet and dry sets) used in this study.**
(XLSX)
