# Supplementary figures and images for: The FIGS (Focused Identification of Germplasm Strategy) Approach Identifies Traits Related to Drought Adaptation in Vicia faba Genetic Resources
Source: PLoS One. 2013 May 8;8(5):e63107. doi: 10.1371/journal.pone.0063107 (PMC3648475; doi:10.1371/journal.pone.0063107)

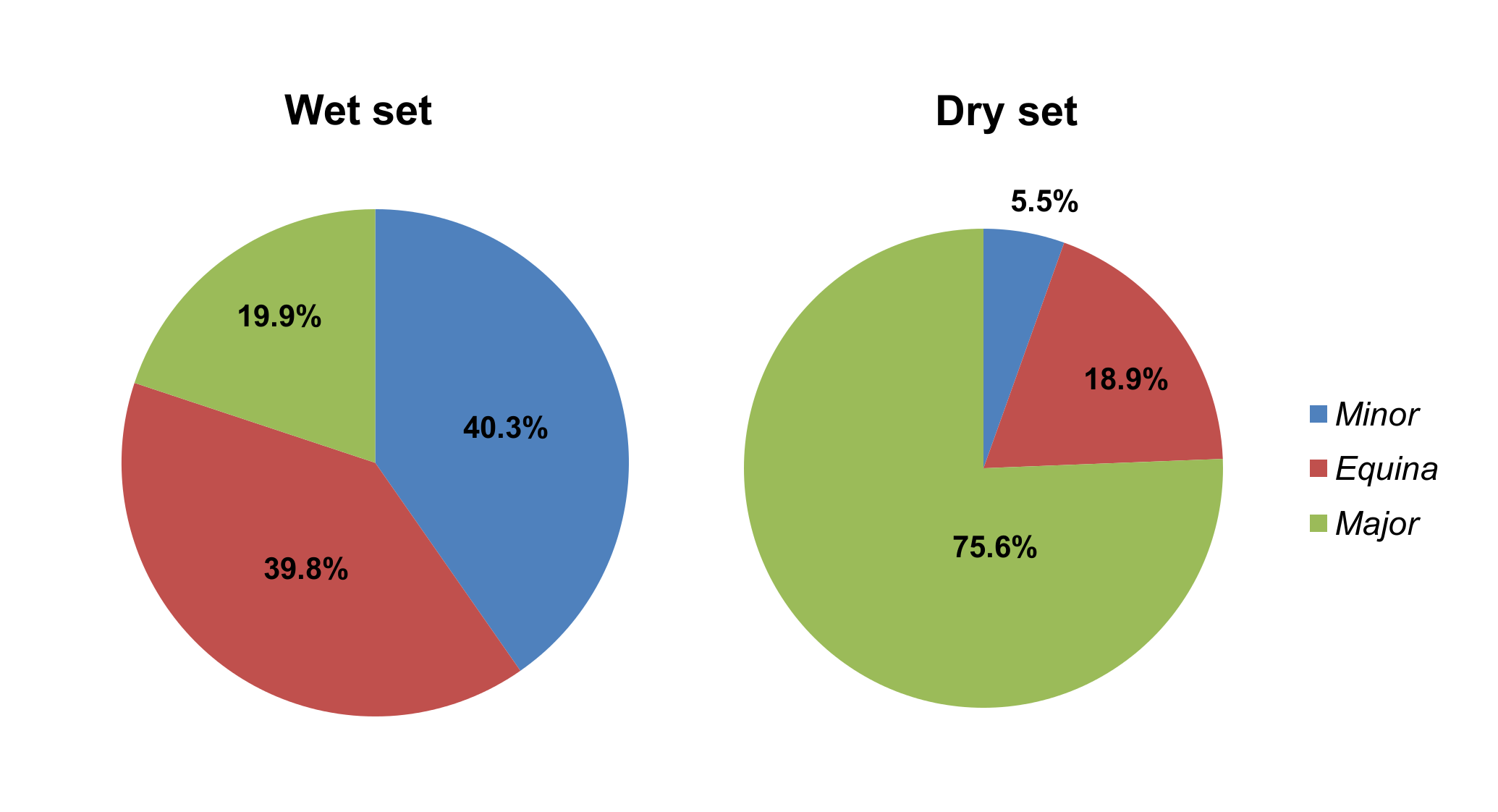

Supplement: Figure S1 — Distribution of seed size classes ( minor , equina and major ) among wet and dry set germplasm. (TIF) [file pone.0063107.s001.tif]
